# Assessment of wall-shear stress pre and post renal sympathetic nerve denervation in patients with resistant hypertension

**DOI:** 10.1186/1532-429X-17-S1-Q60

**Published:** 2015-02-03

**Authors:** Adelina Doltra, Arthur Hartmann, Leonid Goubergrits, Titus Kuehne, Philipp Stawowy, Rolf Gebker, Christopher Schneeweis, Stephan Dreysse, Bernhard Schnackenburg, Eckart Fleck, Sebastian Kelle

**Affiliations:** 1 German Heart Institute Berlin, Berlin, Germany; 2 Biofluid Mechanics Laboratory, Charité Universitätsmedizin Berlin, Berlin, Germany; 3 Clinical Science, Philips Healthcare, Hamburg, Germany

## Background

Renal denervation (RD) is a promising treatment for patients with resistant hypertension, with a good safety profile. Studies performed in animal models have demonstrated an increase in renal arteries' velocity and flow. The hemodynamic effects of RD on renal arteries in humans remain unknown. The aim of our study is to evaluate the hemodynamic effect of RD on renal arteries non-invasively, using magnetic resonance (MR) techniques.

## Methods

17 patients (age 65 ± 7.5 years, 28% women) with resistant hypertension undergoing RD were included. A 3.0 Tesla MR study (Ingenia, Philips Healthcare, The Netherlands) was performed before RD and 6 months after, and flow measurements of both renal arteries were obtained using through-plane breath-hold phase-contrast MR imaging. Peak velocity and mean flow were calculated. In addition, in a subset of 10 patients wall shear stress of both renal arteries was calculated using computerized flow analysis.

## Results

Systolic blood pressure decreased significantly after RD (149 ± 17 (147) mmHg baseline vs. 142 ± 16 (139), p = 0.01). A significant increase in peak velocity (656.72 ± 178.86 (620) mm/s baseline vs. 767.53 ± 301.55 (704) mm/s at follow-up, p = 0.021), and mean flow (5.8 ± 2.84 (5.6) ml/s baseline vs. 7.1 ± 3.48 (6.3) ml/s at follow-up, p = 0.007) was observed at 6 months follow-up. The computerized flow analysis demonstrated a significant decrease in wall shear stress in comparison to baseline (1.87 ± 1.23 Pa baseline vs. 1.39 ± 0.78 Pa at follow-up, p = 0.029). The observed results were independent of blood pressure reduction with RD.

In the Figure, an example of wall shear stress is shown. As shown in the scale, red areas represent higher wall shear stress regions. The extent of this areas decreases 6 months after renal denervation. In addition, enlargement of the renal arteries can be documented, indicating a reduced vascular sympathetic tone.

**Figure 1:**
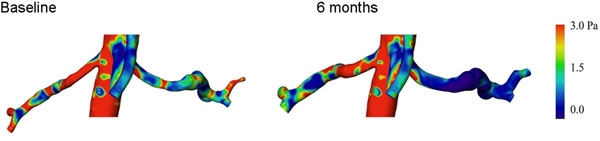


## Conclusions

RD leads to an increase in renal arteries' peak velocity and flow, as well as a concomitant decrease in wall shear stress. These effects reflect the disruption of sympathetic stimuli to the renal arteries with RD, independent of blood pressure response.

## Funding

A. Doltra was supported by a Research Grant from the European Society of Cardiology.

